# A retrospective qualitative report of symptoms and safety from transcranial focused ultrasound for neuromodulation in humans

**DOI:** 10.1038/s41598-020-62265-8

**Published:** 2020-03-27

**Authors:** Wynn Legon, Sarah Adams, Priya Bansal, Parantap D. Patel, Landon Hobbs, Leo Ai, Jerel K. Mueller, Gregg Meekins, Bernadette T. Gillick

**Affiliations:** 1 Division of Physical Therapy and Rehabilitation Science, Department of Rehabilitation Medicine, School of Medicine, University of Minnesota, MN Minneapolis, USA; 2 Department of Neurological Surgery, School of Medicine, University of Virginia, VA Charlottesville, USA; 3 School of Medicine, University of Virginia, VA Charlottesville, USA; 4 Department of Neurology, School of Medicine, University of Minnesota, MN Minneapolis, USA

**Keywords:** Neuroscience, Neurology, Risk factors

## Abstract

Low intensity transcranial focused ultrasound (LIFU) is a promising method of non-invasive neuromodulation that uses mechanical energy to affect neuronal excitability. LIFU confers high spatial resolution and adjustable focal lengths for precise neuromodulation of discrete regions in the human brain. Before the full potential of low intensity ultrasound for research and clinical application can be investigated, data on the safety of this technique is indicated. Here, we provide an evaluation of the safety of LIFU for human neuromodulation through participant report and neurological assessment with a comparison of symptomology to other forms of non-invasive brain stimulation. Participants (N = 120) that were enrolled in one of seven human ultrasound neuromodulation studies in one laboratory at the University of Minnesota (2015–2017) were queried to complete a follow-up Participant Report of Symptoms questionnaire assessing their self-reported experience and tolerance to participation in LIFU research (I_sppa_ 11.56–17.12 W/cm^2^) and the perceived relation of symptoms to LIFU. A total of 64/120 participant (53%) responded to follow-up requests to complete the Participant Report of Symptoms questionnaire. None of the participants experienced serious adverse effects. From the post-hoc assessment of safety using the questionnaire, 7/64 reported mild to moderate symptoms, that were perceived as ‘possibly’ or ‘probably’ related to participation in LIFU experiments. These reports included neck pain, problems with attention, muscle twitches and anxiety. The most common unrelated symptoms included sleepiness and neck pain. There were initial transient reports of mild neck pain, scalp tingling and headache that were extinguished upon follow-up. No new symptoms were reported upon follow up out to 1 month. The profile and incidence of symptoms looks to be similar to other forms of non-invasive brain stimulation.

## Introduction

Transcranial focused ultrasound (LIFU) is a new and promising non-surgical low-energy technique that uses mechanical energy to modulate neuronal activity with high spatial resolution and adjustable depth of focus. LIFU has been used safely and effectively for cortical and sub-cortical neuromodulation in mouse^1–4^, rat^5–7^, rabbit^8^, sheep^9,10^, pig^11^ and primate^12–15^, and has also been demonstrated to be an effective method of transient cortical and sub-cortical neuromodulation in humans^16,17^. In humans, LIFU has been applied to the temporal cortex^18^, primary somatosensory cortex (S1)^16,19^, secondary somatosensory cortex (S2)^20^, primary motor cortex^21^, primary visual cortex^22^ and thalamus^17,23^. LIFU has been shown to affect the amplitude of evoked potentials^8,16,19^, the power, phase and frequency of the electroencephalogram (EEG)^16,24^; the blood oxygen level dependent (BOLD) magnetic resonance imaging signal^8,25,26^, as well as tactile^16,19^ and reaction time^21^ behavior.

As currently employed, human neuromodulation with LIFU typically involves coupling one (or more than one^20^) focused single-elements usually in the ~250 to 600 kHz range (for efficient energy transfer through skull^27^) to the scalp to target a desired brain region. Transducers for cortical targeting are generally small (~30 mm diameter with a ~30 mm focal length); produce a ~3–4 millimeter lateral and ~1–2 centimeter axial resolution, and can be placed anywhere on the head similar to other current methods of non-invasive brain stimulation. Ultrasound is also capable of reaching brain targets deep to the cortical surface as the acoustic waves can be focused to any desired depth within certain physical limits of the transducer employed. Transducers for deep brain modulation are typically larger (~70 mm diameter)^17,23^ to achieve this deeper focal length at reasonable axial resolutions^17^. In addition to adjusting focal lengths, there are a number of parameters that can be manipulated when using ultrasound including the acoustic frequency, amplitude, duration, duty cycle, pulse repetition frequency etc. and the efficacy of some of these for successful neuromodulation has been addressed in small^3,4^ and large animal studies^10^ though the mechanism of acoustic energy for neuronal modulation is largely theoretical^28–31^ and the impact of parameter space in humans is not yet well-described. The bioeffects of ultrasound for neuromodulation in humans as described here are likely largely mechanical as opposed to thermal or cavitational as the parameters used are of low intensity and short duration and generate temperatures insufficient for thermal modulation^32^. Ultrasound for transient neuromodulation is different from the use of ultrasound for surgery where high intensities are used to thermally ablate tissues^33,34^ or for transient blood-brain barrier (BBB) opening where high intensities are also used in combination with contrast agents (microbubbles) to intentionally produce cavitation as a means of opening the BBB^35,36^.

In its current state, ultrasound for neuromodulation generally follows the safety guidelines of the Food and Drug Administration (FDA) for obstetric diagnostic ultrasound and adult cephalic applications^37^. These include derated limits of spatial peak pulse average (I_sppa_) of 190 W/cm^2^, a spatial peak temporal average of 720 mW/cm^2^ (94 mW/cm^2^ for adult cephalic) and a mechanical index (MI = peak negative pressure/√fcenter frequency) of 1.9. MI is an indication of the ability to produce cavitation related bio-effects and can be used as an indication for potential micromechanical damage. In addition to FDA guidelines, the IEC 60601 part 2 standard for therapeutic equipment sets 3 W/cm^2^ as the limit on acoustic intensity^38^. These levels of energy are generally respected in human ultrasonic neuromodulation studies^16,17,19–22,24–26^ even though there are no definitive guidelines for energy deposition into the human brain. There is a long history of ultrasound for diagnostic and therapeutic applications, but explicit expository and dosimetry are still largely lacking^39,40^. There are several thorough reports examining the effect different intensities of ultrasound to affect tissue^41,42^ and efforts made to develop thresholds for potential hazards^43,44^ though these studies typically use continuous wave schemes and high intensities well beyond the levels used for transient neuromodulation and therefore are not wholly informative for low intensity applications. As such, it is important to assess the safety of ultrasound for human neuromodulation. It is the purpose of this paper to provide an initial assessment of the symptoms and minor adverse events and safety of single element focused ultrasound for human neuromodulation as there is little research on participant perceived tolerance and report of symptoms. Here, we report on the findings of a variant of the Participant Report of Symptoms questionnaire^45,46^ assessing participants’ perceived tolerance to participation in LIFU and their perceived relation of any symptoms to the ultrasound intervention. Of a group of 120 queried, a total of (N = 64) consented to completing the questionnaire at various time points from immediately post-experiment out to 22 months.

## Results

### Follow-up response rate

**Figure 1:**
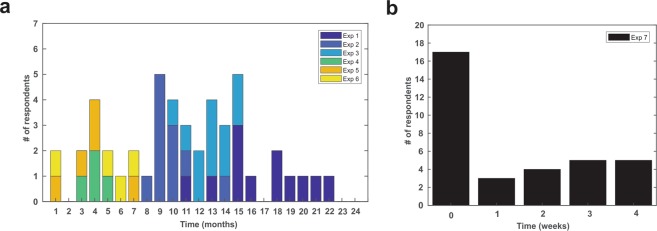
Timeline of respondent follow-up. Bar graph (**a**) of the time of questionnaire response of participants from experiments one through six (N = 47) broken down by experiment number. (**b**) Bar graph of response time for experiment 7. Seventeen participants took the questionnaire the day of the experiment (Time = 0) and responded to the questionnaire again at one of four time points out to one month.

A total of 64/120 (53.3%) participants responded to the email regarding follow-up questionnaire. The mean age of the participants was 22.96 ± 2.14 years (29 Male, 35 Female). See Table 1 for individual experiment demographics and follow-up response rates for each study. The time of response post experimental participation ranged from 1 month to 22 months after participation for experiments 1–6 (see Fig. 1a). For experiment 7, 17 participants responded to the questionnaire immediately post experiment and then were contacted at a random time post experiment out to one month (Fig. 1b).

**Table 1:** Study characteristics and demographics. **not published.

Study	Target	Results	Methods	N	Response	Age (Mean ± SD)	M/F	Response Rate
1^17^	Thalamus	P14 SEP ↓ α, β, γ power ↓	EEG, SEP	20	12	23.4 ± 1.8	5/7	60%
2^17^	Thalamus	Tactile discrimination ↓	Behavior Assessment	20	11	22.7 ± 2.5	5/6	55%
3^21^	M1	Reaction Time ↓	Behavior Assessment	25	11	23.4 ± 1.9	6/5	44%
4^26^	M1	BOLD volume ↓	7 T MRI	6	4	22.5 ± 2.4	3/1	67%
5^21^	M1	MEP amplitude ↓	Single pulse TMS	12	5	24.5 ± 1.0	2/3	42%
6^21^	M1	SICI–, SICF ↓	Paired Pulse TMS	10	4	23.8 ± 1.5	2/2	40%
7^**^	M1	Motor Learning–	EEG, EMG, Behavior	27	17	21.2 ± 2.2	6/11	63%
**Total**				**120**	**64**	**22.96 ± 2.14**	**29/35**	**53.3%**

### Symptoms reported

**Figure 2:**
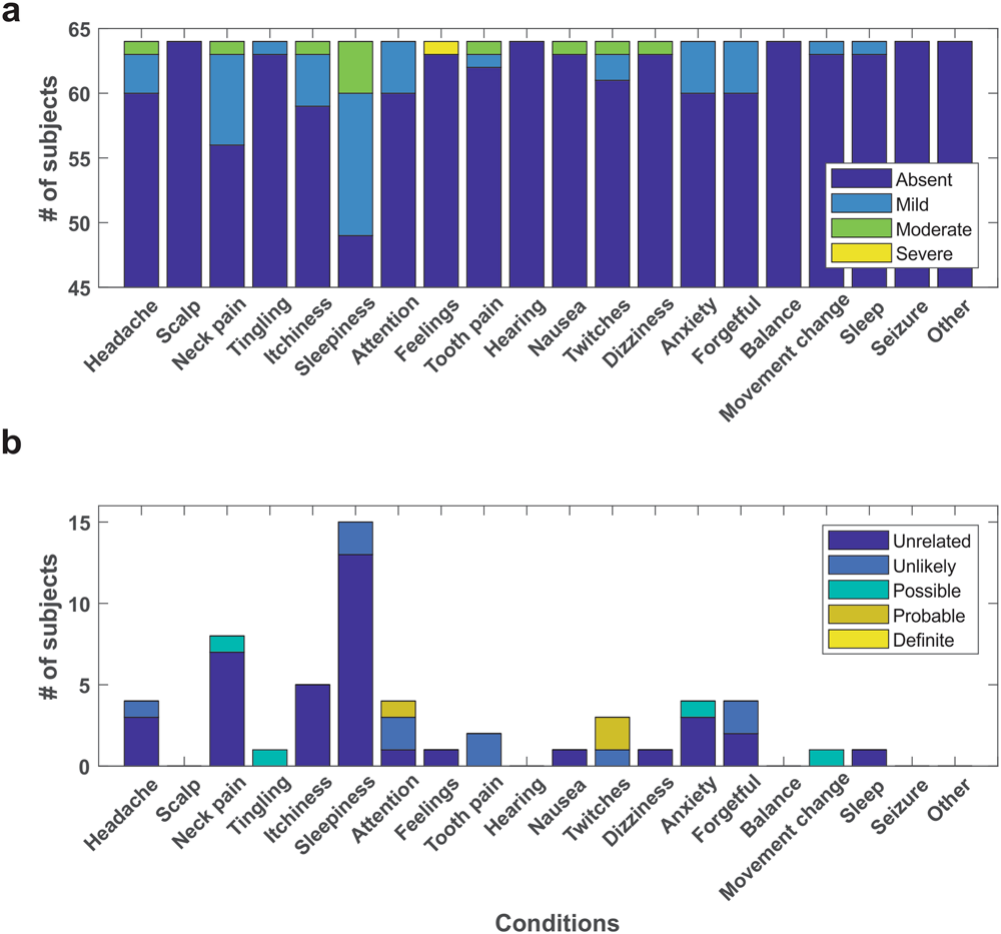
Group report of symptoms. (**a**) Total number of responses for all participants (N = 64) collapsed across all experiments (1–7) coded by the severity of the symptom. (**b**) Total number of responses from all participants (N = 64) collapsed across all experiments coded by the subjective relation of the symptom to the ultrasound neuromodulation intervention. Note: Feelings refers to the question asking about experiencing any unusual feelings, attitudes or emotions.

**Figure 3:**
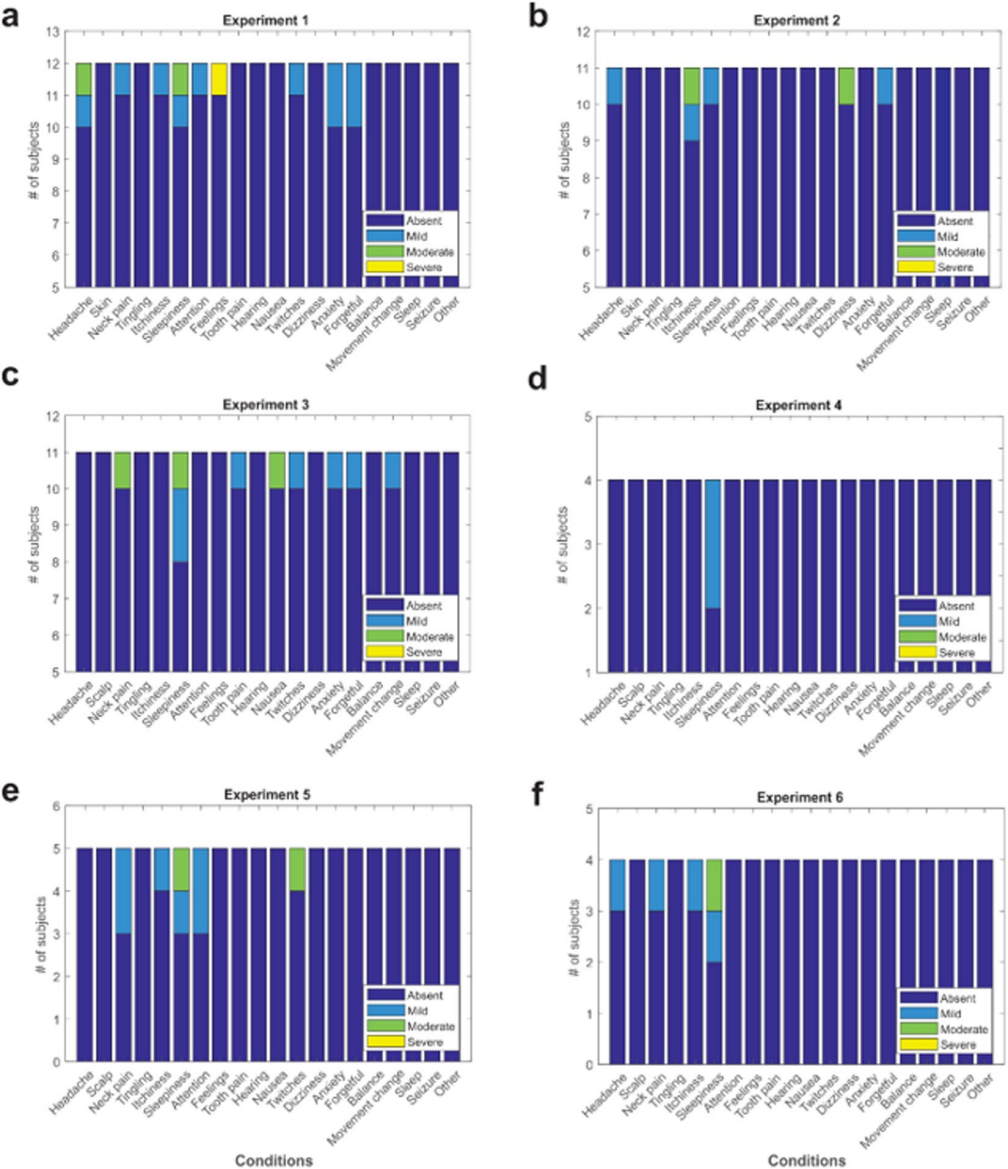
Individual report of symptoms for experiments 1–6.

Data from all seven experiments revealed 7/64 reported mild or moderate symptoms that they felt were ‘possible’ or ‘probably’ related to the ultrasound intervention. These included neck pain, difficulty paying attention, muscles twitches and anxiety. There were no reports of any severe and related symptoms (Fig. 2a). There was however, one report of severe unusual feelings, attitudes or emotions though was reported as being unrelated to LIFU in experiment 3. Of the other reported conditions, participants rated these as unrelated or unlikely related to the ultrasound intervention (see Fig. 2b). The most common reported symptom was sleepiness though this was rated as unrelated or unlikely for all instances. Other responses included headache (n = 4), itchiness (n = 5), tooth pain (n = 1) and forgetfulness (n = 4). No participant rated any reported symptom as definitely related to the ultrasound intervention (see Fig. 2b). See Fig. 3 for a breakdown of symptoms by experiment for experiments 1–6.

### Duration of symptoms

**Figure 4:**
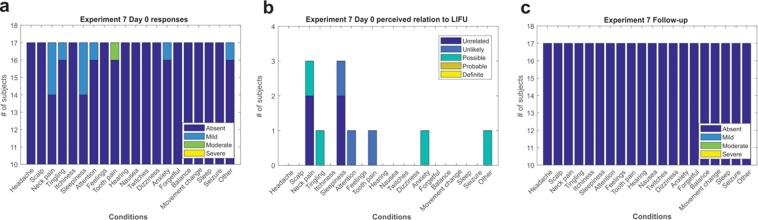
Experiment 7 report of symptoms. (**a**) Report of symptoms immediately after completion of ultrasound experiment. (**b**) Perceived relation of immediate symptom to the ultrasound intervention. (**c**) No new or persistent symptoms were reported at follow-up.

In a subset of participants (n = 17) for experiment 7 we collected response to the questionnaire at two time points: immediately following experimentation (~20 minutes) and then at a randomly assigned follow-up at 1 week, 2 weeks, 3 weeks or 1 month (see Fig. 1b). On day zero, there were three reports of neck pain, three reports of sleepiness, one report of scalp tingling, one report of tooth pain, one report of difficulty paying attention and one report of feeling anxious, worried or nervous and one ‘other’ report of mild back pain (see Fig. 4a,b). No participant reported more than one symptom at initial inquiry. Of these reports, neck pain was perceived as unrelated in two instances and possible in one. Sleepiness was perceived as unrelated in two instances and unlikely in the other. Tingling of the scalp was perceived as possibly related to the intervention, difficulty paying attention was unlikely, tooth pain was unlikely and anxiousness was perceived as possibly related (Fig. 4). At follow-up (1 week to 1 month) these participants did not report any persisting or new effects (Fig. 4c). Of the 7 participants who did not report any initial symptoms, none reported additional symptoms at follow-up (Fig. 4c).

### Correlation of symptom response to LIFU parameters

**Figure 5:**
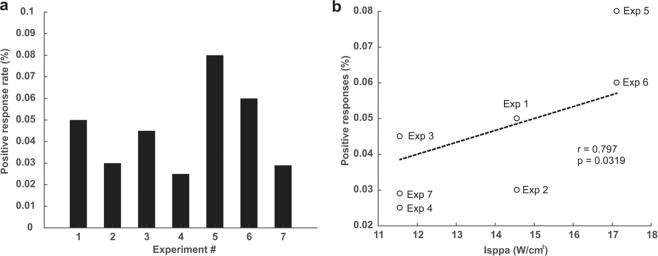
Symptom response rates across experiments. (**a**) Symptom response rate for each of the seven experiments regardless of perceived relation to the intervention. (**b**) Relation of the response rate to the intensity (I_sppa_).

To gauge the overall positive symptom rate, we tabulated all positive responses regardless of subjective report on the relation to the experimental intervention. The overall positive report of symptoms for all experiments (1–7) included in our neurological questionnaire was 55/1280 total possible positives for an overall positive response rate of 4.3%. Of the 55 total positive responses 38/55 (69%) were judged by the participants to be unrelated to the LIFU interventions, 10/55 (18%) unlikely, 4/55 (7%) possible, 3/55 (5%) probable and 0/55 definitely related. The positive response rates for experiments 1–7 were: 5.4%, 3.2%, 4.5%, 2.5%, 8%, 6.3% and 2.9% respectively (see Fig. 5a). As a means to begin to relate ultrasound to potential symptomology we performed correlations between ultrasound parameters and the overall response rate. The linear correlation of the response rate percentage and mechanical index (MI) was not significant (r = 0.633, p = 0.13), there was no linear correlation between number of stimulations and response rate; r = 0.285, p = 0.53 however, (I_sppa_) was found to have a significant positive correlation with response rate; r = 0.797, p = 0.0319 (Fig. 5b).

### Comparison to other non-invasive neuromodulation modalities

A total of 948 subjects from repetitive transcranial magnetic stimulation (rTMS) studies, 83 subjects from deep TMS (dTMS), and 554 subjects from transcranial direct current stimulation (tDCS) studies were included in the overall symptom incidence calculations. References for the included papers in this analysis are provided in Supplementary Materials. Only those incidences that could be directly compared (had a similar label or categorization to those used in our questionnaire) to the LIFU symptom incidences from this study are presented below except for the dTMS. The most common symptoms from rTMS included headache (14.6%) and neck pain (3.8%); tDCS reported itchiness (16.6%), tingling (13.2%), and sleepiness (10.8%) most frequently. Ultrasound had no incidences of seizure and lower incidence of headache than both forms of TMS, but higher incidences of neck pain, attention issues, twitching, tingling, itchiness, and sleepiness. Compared to tDCS, ultrasound had similar incidences of headache, attention issues and feelings of nausea and both have no report of seizure. A comparison of symptom profiles is summarized in Table 2. Because ultrasound is capable for sub-cortical neuromodulation, comparison of reported symptoms to dTMS is highly relevant if one is to consider either LIFU or dTMS for sub-cortical neuromodulation. Deep TMS is a method of TMS that uses specific coil geometries to help penetrate and focus the magnetic field deeper into the brain for stimulation of sub-cortical targets^47^. From our literature review, we found a similar total number of subjects (N = 83) for dTMS as reported here. The most common reported symptom(s) from dTMS were general discomfort and/or unpleasantness (21.7%) and headache (10.8%). A comparison of symptom profiles is shown in summary Table 2. One of the feasibility and safety dTMS studies published was not included in the summary above since the number of symptom occurrences were not reported^48^. It was reported that some dTMS (H1-coil, H2-coil) subjects experienced dizziness but rTMS and sham TMS subjects did not. Perhaps more strikingly, some H1-coil dTMS subjects experienced feelings of detachment and a transient impairment in spatial recognition memory and Lenoir *et al*. (2018) recently reported two cases of seizure using dTMS for insular stimulation^49^.

**Table 2:** Neuromodulation comparison of symptoms.

	LIFU	rTMS	dTMS	tDCS
*Number of Subjects*	64	948	83	554
Age Range	18–38	18–65	20–54	18–73
% Female	55.0%	45.4%	48.8%	45.5%
% Reported during session	21.0%	97.2%	100.0%	100.0%
% Reported at a follow up	79.0%	6.8%	27.5%	6.8%
Headache	6.3%	14.6%	10.8%	6.9%
Neck Pain^a^	12.5%	3.8%	3.6%	NR
Nausea	1.6%	1.4%	NR	0.9%
Attention Issues^b^	6.3%	NR	NR	8.1%
Seizure	0.0%	0.3%	2.4%	NR
Twitching^c^	4.7%	1.7%	NR	NR
Tingling	1.6%	NR	NR	13.2%
Itchiness^d^	7.8%	NR	NR	16.6%
Sleepiness^e^	23.4%	0.1%	5.2%	10.8%
Discomfort	NR	6.98%	21.7%	3.61%

^a^Neck Pain: includes reported neck pain, neck ache, pain in trigeminal nerve region, or jaw ache.

^b^Attention Issues: includes reported attention issues or difficulty concentrating.

^c^Twitching: includes twitching, muscle twitching, or muscular problems.

^d^Itchiness: includes reported itchiness, itching, itching sensation, or contact dermatitis.

^e^Sleepiness: includes reported sleepiness, fatigue, or tiredness.

NR = not reported.

## Discussion

In this report, we provide a qualitative assessment of minor adverse events reported from seven experiments conducted in our lab using single element LIFU for human neuromodulation. We collected retrospective data via a participant report of symptoms questionnaire administered over the telephone at varying time points post experiment that ranged from 0 months (day of experiment collected in person) to 22 months post experiment (telephone interview). 64/120 total participants responded to the questionnaire. Symptoms included headache, neck pain, itchiness, sleepiness, problems with attention, tooth pain, muscle twitches, and anxiety. Only one of these reports was rated as severe and none were reported as definitely related to the LIFU intervention. A subset of participants took the questionnaire immediately after experimentation. Immediate symptoms included mild headache, mild neck pain, and tingling in the scalp. None of these symptoms persisted upon follow-up out to one month and no new symptoms were reported. The intensity (I_sppa_) of ultrasound ranged from 11.56 W/cm^2^ to 17.12 W/cm^2^ (in free water) for the experiments included in this study and we found a significant positive linear correlation of the symptom response rate and the ultrasound intensity (I_sppa_). The intensity (I_sppa_) used in these studies is considerably lower than FDA thresholds for ultrasound diagnostics. Despite a lack of definitive causation, and the finding that most of the reported symptoms were believed by the participants to be unrelated to the LIFU intervention, this finding nevertheless speaks to limiting the intensity used in future ultrasound experiments and determining as low as reasonably achievable levels for neuromodulation. Despite the I_sppa_ level being below FDA thresholds, the I_spta_ (spatial peak temporal average) in these studies was above FDA thresholds for diagnostics. I_spta_ is defined by the I_sppa_ multiplied by the duty factor providing a metric of the average intensity over the duration of the pulse. One of the main concerns of determining safe intensity levels is estimating the intracranial pressure. The intensities presented here were taken from empirical recordings in free water and hence the derated intensities will be considerably lower. The skull is highly attenuative to ultrasound and the *in situ* derated pressures are not exactly known but can be estimated using either empirical pressure measurements using a hydrophone through skull fragments or through computer modelling that takes into consideration the acoustic properties of bone and tissue^16,22,50^. For low intensity neuromodulation, in general, ultrasound intensity intracranially is estimated to be attenuated ~3–4 fold from values measured in free water^16^ and would thus produce I_spta_ values under the FDA recommended limits for obstetric diagnostics (720 mW/cm^2^)^37^.

The experiments documented here had a rather small range of I_sppa_ though used considerably different number of stimulations and different inter-stimulus intervals that would contribute to overall exposure and may influence potential hazard. Indeed, Lee *et al*. (2016) found that a high number of total stimulations (600) with a low ISI (1 s) resulted in evidence of microhemorrhage in sheep despite the intensity being rather low at 6.6 W/cm^2,9^. We found no relation of number of stimulations with symptom prevalence though there were other variables that were also manipulated such as the inter-stimulus interval. It is currently unclear what constitutes a high number of total stimulations or a low ISI though taking these experimental parameters into consideration in the planning of experimental design is prudent. Additional metrics like energy density (J/cm^2^) as has been used in ultrasound sonoporation^51^ and neuromodulation parameter^4^ studies, as well as the total experimental energy density (J/cm^2^) that takes into account intensity, duty cycle as well as total number of stimulations and the ISI would prove an additional valuable safety metric given the results of Lee *et al*.^9^ though the relation of total experimental energy density to hazard is not well understood.

In addition to our group, Seung-Schik Yoo’s lab has performed multiple human ultrasound neuromodulation studies and completed thorough safety analysis including similar telephone follow-up as well as neurological assessment pre and post experiment including anatomical MRI and reported zero events in their three studies^19,20,22^. In the published literature and including the experiments in this study, a total of 260 individuals have participated in human ultrasound neuromodulation experiments to date with no reported serious adverse events and the data from this report is the first to report on minor transient events associated with the LIFU intervention. Caution is always advised when imparting energy into the brain and further research is recommended examining the effect of total number of stimulations and the inter-stimulus interval and the potential interaction of these parameters with intensity and duty cycle. Consideration of these parameters should be undertaken in experimental design to keep total experimental energy levels as low as reasonably achievable. Despite the differences in total number of stimulations and ISI in the experiments reported here, there were no large differences in response rate or in the type of report of symptoms between the seven experiments. It should be noted here too, that the experiments included in this report used additional technologies such as EEG, MRI and TMS in addition to LIFU that could potentially account for some of the positive findings. To this point, in experiments that employed TMS in addition to LIFU, we cannot tease out if symptoms were related to TMS or LIFU interventions. However, the fact that there isn’t a large difference in total symptoms for these studies as compared to the studies that just used LIFU suggests that there are not compounding or additive symptomology with concurrent forms of neuromodulation. To provide additional context to the safety assessments of ultrasound neuromodulation, we have compared our experiments’ symptom response rate and symptom profile to other forms of non-invasive human neuromodulation, including repetitive transcranial magnetic stimulation (rTMS), deep transcranial magnetic stimulation (dTMS), and transcranial direct current stimulation (tDCS).

The overall symptom response rate and type of symptoms is similar but without follow-up with all participants, it is possible that some additional symptoms might have occurred and were not included in the analysis. It should be noted here however, that our comparative analysis has some significant limitations. Firstly, we utilized highly restrictive exclusion criteria in our literature review. By excluding protocols that failed to report at least one event following neuromodulation, the calculated incidences presented may be greater than expected. Secondly, because we aimed to calculate overall incidences for symptoms across subjects, we excluded several studies that provided incidences across treatment sessions, further limiting the data. Thirdly, this study was one of the few that described the extent to which subjects attributed symptoms to the treatment itself though participant subjective assessment of the relation of symptoms to the intervention is speculative. It would be prudent for future studies to include physical examinations by a physician at multiple regular intervals to clarify patterns of symptoms for safety guidelines.

## Conclusions

We provide qualitative data of reported symptoms from seven separate experiments from a single research team using different experimental protocols and energy levels of ultrasound for human neuromodulation as assessed by participant report of symptom questionnaire. Symptom rate and type were similar across studies and are similar to other forms of human non-invasive neuromodulation like TMS and tDCS that have a long-standing history of being safe forms of human neuromodulation.

## Material and methods

### Participants

All experiments were conducted with the approval of the Institutional Review Board at the University of Minnesota. All research was performed in accordance with relevant guidelines/regulations and all participants provided written informed consent to participate. The data presented here is from a one laboratory. A total of 120 volunteer study participants (48 male, 72 female aged 18–38 with a mean age of 22.96 ± 2.14 years) provided written informed consent to participate in one or more of the seven experiments from which the data for this study is taken between 2015 and 2017 at the University of Minnesota. Prior to formal experimental procedures, participants were screened via questionnaire for contraindications to non-invasive neuromodulation and none of the participants reported any neurological impairment or identified any contraindications to non-invasive neuromodulation as outlined by Rossi *et al*. (2009) as identified for transcranial magnetic stimulation^52^.

### Experiments

The data for this study is a summary of 64 individual participants that participated in one of seven completed experiments conducted in our lab in the Department of Rehabilitation Medicine at the University of Minnesota. Details of the specific objectives or hypotheses of each study are not elaborated upon though it was the purpose of all studies to assess how LIFU targeting either the primary motor cortex or the thalamus affected physiological markers of neuronal activity and behaviour. Specifics of the methods can be found in Legon *et al*. (2018a) and Legon *et al*. (2018b). We have included details on the LIFU application including transducer specifics, target (cortical, sub-cortical) and parameters (amplitude, duration, etc.) are enumerated. For the purposes of this report, experiments will be referred to by number (1–7) based upon chronological date of commencement. All experiments were conducted in neurologically healthy volunteer participants to test the effect of LIFU on either cortical or sub-cortical neuronal excitability and/or effect on specific behaviours. The environment of the experiments differed as one experiment (Experiment 4)^26^ was conducted in a 7 T MRI scanner at the Center for Magnetic Resonance Research at the University of Minnesota (https://www.cmrr.umn.edu) and experiment 6 and 7 also involved transcranial magnetic stimulation (TMS) either concurrent with LIFU or as a pre/post measure of motor cortical excitability^21^. For all experiments (except fMRI experiment), participants were seated in a dentist-type chair and asked to either perform a simple task or sit passively for the duration of the experimental protocol. Tasks included a sensory discrimination task^16^ and simple stimulus response tasks on a computer. All experiments were repeated measures design with either one of or both an active or passive sham along with the LIFU condition. See Table 1 for experiment recruitment totals, participant demographics, and cited studies for individual experimental design and targeting details.

### Questionnaire and follow-up

For all experiments, participants were retrospectively contacted via email at random time intervals (1 week–22 months) post experiment for their willingness to participate in a follow-up questionnaire on their experience of undergoing LIFU neuromodulation and perceived relation of any reported symptoms to LIFU. For all experiments, participants were contacted via email only once. For experiment 7, participants filled out the questionnaire immediately (~20 minutes after LIFU): (This is designated as 0 months in Figures and following text) after experimentation with follow-up at one of four time points post-experiments (1 week, 2 weeks, 3 weeks and 1 month). Those participants who responded affirmatively via email were subsequently contacted via telephone and asked to respond to 20 questions regarding their subjective assessment of their current neurological health (see Supplementary Material for questionnaire). This questionnaire is a variant of the Participant Report of Symptoms questionnaire that has previously been used in other non-invasive neuromodulation studies^45,46^. If there was a positive response to a question indicating perceived experience of the symptom, participants were then asked to rank the symptom severity from 2–4 (1 = absent) where 2 = mild, 3 = moderate and 4 = severe. In addition, participants were asked for their subjective assessment of the relation of the symptom to their involvement in the ultrasound experiments. Potential responses were: 1 = unrelated, 2 = unlikely, 3 = possible, 4 = probable and 5 = definite. In instances of positive subjective report – each case was referred to a neurologist (G.M) for medical record review and assessment of reported symptoms. Participants were remunerated for their participation in this telephone interview session. Total phone call discussion time ranged from 5–10 minutes. All phone calls were conducted by one of two lab investigators.

### Transcranial focused ultrasound

For all experiments, the LIFU condition involved acoustically coupling the active face of the ultrasound transducer to the scalp at the pre-determined site depending upon the target of interest. Targeting was done using individual participant structural MRI scans paired with a neuronavigation system (Brainsight, Rogue Research). neuronavigation was used for localization for LIFU targets. The safety and comfort of participants was monitored through self-report during LIFU. No cavitation monitoring or temperature measurements were completed for these experiments The passive sham condition involved either placing a high acoustic impedance disk on the face of the transducer (Experiments 1 and 2)^17^, flipping the transducer over (while on) or simply turning it off during collection (experiment 4 MRI)^26^. For active sham conditions, ultrasound was delivered to another brain region (Experiment 3)^21^. Shamming maintained contact of the transducer to the head to mimic the audible sensation of a slight buzzing but attenuate any energy into the head. The audible sound was identical for sham and LIFU conditions and no subjects reported any sensory or perceptual differences between sham and LIFU conditions as previously reported^16^. This identical audible sound for sham and LIFU was designed to mitigate potential effects of indirect activation of auditory pathways^53,54^. The active sham condition, when employed, involved delivering LIFU to another scalp site (vertex) with the same parameters as the experimental site^21^. All experiments employed a repeated measures design where the participant received LIFU and sham stimulation in the same session regrettably making a disassociation of symptoms between real and sham stimulation not possible.

### LIFU waveforms

All experiments used a single element 0.5 MHz transducer. Transcranial ultrasonic neuromodulation waveforms were generated using a two-channel, 2-MHz function generator (BK 4078B Precision Instruments). Channel 1 was used to gate channel 2 that was a 500 kHz sine wave. Channel 1 is the pulse repetition frequency (PRF) and was a 5Vp-p square wave burst of 1 kHz (N = 500) resulting in a 0.5 second total duration. The duty cycle was 36%; the period is 1/PRF resulting in a single pulse duration of 360 µs. The output of channel 2 was sent through a 100-W linear RF amplifier (E&I 2100 L; Electronics & Innovation) before being sent to the custom-designed focused ultrasound transducer. A total of 3 different transducers were used across the 7 experiments. Experiments 1 and 2 used the same transducer; experiments 3, 4 and 7 used the same transducer and experiments 5 and 6 used the same transducer. See Table 3 for ultrasound beam parameters and focality, and Table 4 for transducer specifications, including intensity and peak negative pressure for each study.

**Table 3:** Transducer characteristics.

Experiment	Af (kHz)	Aperture (mm)	Focal length (mm)	F#	Beam Geometry (−3dB)		
					**X (mm)**	**Y (mm)**	**Z (mm)**
1	500	63	71	1.13	3.6	3.6	19.6
2	500	63	71	1.13	3.6	3.6	19.6
3	500	30	24	0.8	1.8	1.7	25.2
4	500	30	24	0.8	1.8	1.7	25.2
5	500	30	25	0.83	2.1	2.2	25.4
6	500	30	25	0.83	2.1	2.2	25.4
7	500	30	24	0.8	1.8	1.7	25.2

**Table 4:** Neuromodulation parameters.

Experiment	# of stimulations	ISI (sec)	I^sppa^ (W/cm^2^)	I^spta^ (W/cm^2^)	MI
1	300	4	14.56	5.24	0.89
2	90	8	14.56	5.24	0.89
3	200	4	11.56	4.16	0.78
4	54	5.5	11.56	4.16	0.78
5	80	10	17.12	6.16	0.9
6	150	10	17.12	6.16	0.9
7	44	4.75	11.56	4.16	0.78

### Comparison of LIFU symptoms to other non-invasive brain stimulation modalities

To better assess and relate the reported symptoms from LIFU we compared our results to reported symptoms from other non-invasive brain stimulation modalities including repetitive transcranial magnetic stimulation (rTMS), deep TMS (dTMS) and transcranial electric stimulation (TES). To do so, we performed a literature search on the PubMED database, identifying 22 publications pertaining to rTMS safety in healthy subjects (from 1992 to 2013), 7 publications pertaining to dTMS safety in healthy subject (from 2004 to 2018), and 7 publications pertaining to tDCS safety in healthy subjects (from 2004 to 2015). We utilized the following search criteria: (transcranial magnetic stimulation) AND safety AND Humans; (transcranial magnetic stimulation) AND deep OR H-coil AND Humans; (transcranial direct current simulation) AND safety AND Humans. Articles were excluded if they did not report at least one minor adverse event following neuromodulation; did not include healthy subjects; did not report the total number of healthy subjects or did not report total of incidences of symptoms. Studies focusing on pediatric or geriatric populations were also excluded, as were single patient case studies. Associated article references were also reviewed and included in the analysis if they met the preceding criteria. The number of healthy subjects, incidence of specific symptoms, and pertinent stimulation parameters were obtained from each study. Overall symptom incidences were calculated by summating incidences across all studies for each rTMS, dTMS, and tDCS.

## Supplementary information

Supplementary information.

Supplementary information2.
