# Supplementary material for: A retrospective qualitative report of symptoms and safety from transcranial focused ultrasound for neuromodulation in humans
Source: Sci Rep. 2020 Mar 27;10:5573. doi: 10.1038/s41598-020-62265-8 (PMC7101402; doi:10.1038/s41598-020-62265-8)
Supplement: Supplementary file 1 — Supplementary information. [file 41598_2020_62265_MOESM1_ESM.docx]

**rTMS references**

1. Anderson, B., Mishory, A., Nahas, Z., Borckardt, J. J., Yamanaka, K., Rastogi, K., & George, M. S. (2006). Tolerability and safety of high daily doses of repetitive transcranial magnetic stimulation in healthy young men. *The journal of ECT*, *22*(1), 49-53.
2. Cohrs, S., Tergau, F., Riech, S., Kastner, S., Paulus, W., Ziemann, U., ... & Hajak, G. (1998). High-frequency repetitive transcranial magnetic stimulation delays rapid eye movement sleep. *Neuroreport*, *9*(15), 3439-3443.
3. Flitman, S. S., Grafman, J., Wassermann, E. M., Cooper, V., O'Grady, J., Pascual-Leone, A. M. D. P., & Hallett, M. (1998). Linguistic processing during repetitive transcranial magnetic stimulation. *Neurology*, *50*(1), 175-181.
4. Gerschlager, W., Christensen, L. O. D., Bestmann, S., & Rothwell, J. C. (2002). rTMS over the cerebellum can increase corticospinal excitability through a spinal mechanism involving activation of peripheral nerve fibres. *Clinical Neurophysiology*, *113*(9), 1435-1440.
5. Graf, T., Engeler, J., Achermann, P., Mosimann, U. P., Noss, R., Fisch, H. U., & Schlaepfer, T. E. (2001). High frequency repetitive transcranial magnetic stimulation (rTMS) of the left dorsolateral cortex: EEG topography during waking and subsequent sleep. *Psychiatry Research: Neuroimaging*, *107*(1), 1-9.
6. Grossheinrich, N., Rau, A., Pogarell, O., Hennig-Fast, K., Reinl, M., Karch, S., ... & Padberg, F. (2009). Theta burst stimulation of the prefrontal cortex: safety and impact on cognition, mood, and resting electroencephalogram. *Biological psychiatry*, *65*(9), 778-784.
7. Koch, G., Mori, F., Marconi, B., Codecà, C., Pecchioli, C., Salerno, S., ... & Caltagirone, C. (2008). Changes in intracortical circuits of the human motor cortex following theta burst stimulation of the lateral cerebellum. *Clinical Neurophysiology*, *119*(11), 2559-2569.
8. Koren, D., Shefer, O., Chistyakov, A., Kaplan, B., Feinsod, M., & Klein, E. (2001). Neuropsychological effects of prefrontal slow rTMS in normal volunteers: a double-blind sham-controlled study. Journal of clinical and experimental neuropsychology, 23(4), 424-430.
9. Machii, K., Cohen, D., Ramos-Estebanez, C., & Pascual-Leone, A. (2006). Safety of rTMS to non-motor cortical areas in healthy participants and patients. *Clinical Neurophysiology*, *117*(2), 455-471.
10. Maizey, L., Allen, C. P., Dervinis, M., Verbruggen, F., Varnava, A., Kozlov, M., ... & Hounsell, C. A. (2013). Comparative incidence rates of mild adverse effects to transcranial magnetic stimulation. *Clinical neurophysiology*, *124*(3), 536-544.
11. Menkes, D. L., Bodnar, P., Ballesteros, R. A., & Swenson, M. R. (1999). Right frontal lobe slow frequency repetitive transcranial magnetic stimulation (SF r-TMS) is an effective treatment for depression: a case-control pilot study of safety and efficacy. *Journal of Neurology, Neurosurgery & Psychiatry*, *67*(1), 113-115.
12. Michael, N., Gösling, M., Reutemann, M., Kersting, A., Heindel, W., Arolt, V., & Pfleiderer, B. (2003). Metabolic changes after repetitive transcranial magnetic stimulation (rTMS) of the left prefrontal cortex: a sham‐controlled proton magnetic resonance spectroscopy (1H MRS) study of healthy brain. European Journal of Neuroscience, 17(11), 2462-2468.
13. Mosimann, U. P., Rihs, T. A., Engeler, J., Fisch, H. U., & Schlaepfer, T. E. (2000). Mood effects of repetitive transcranial magnetic stimulation of left prefrontal cortex in healthy volunteers. Psychiatry Research, 94(3), 251-256.
14. Mottaghy, F. M., Hungs, M., Brügmann, M., Sparing, R., Boroojerdi, B., Foltys, H., ... & Töpper, R. (1999). Facilitation of picture naming after repetitive transcranial magnetic stimulation. *Neurology*, *53*(8), 1806-1806.
15. Niehaus, L., Hoffmann, K. T., Grosse, P., Röricht, S., & Meyer, B. U. (2000). MRI study of human brain exposed to high-dose repetitive magnetic stimulation of visual cortex. *Neurology*, *54*(1), 256-256.
16. Pascual-Leone, A., Houser, C. M., Reese, K., Shotland, L. I., Grafman, J., Sato, S., ... & Hallett, M. (1993). Safety of rapid-rate transcranial magnetic stimulation in normal volunteers. *Electroencephalography and Clinical Neurophysiology/Evoked Potentials Section*, *89*(2), 120-130.
17. Pascual-Leone, A., Valls-Solé, J., Brasil-Neto, J., Cohen, L., & Hallett, M. (1992). Seizure induction and transcranial magnetic stimulation. The Lancet, 339(8799), 997.
18. Ragert, P., Camus, M., Vandermeeren, Y., Dimyan, M. A., & Cohen, L. G. (2009). Modulation of effects of intermittent theta burst stimulation applied over primary motor cortex (M1) by conditioning stimulation of the opposite M1. Journal of neurophysiology, 102(2), 766-773.
19. Rami, L., Gironell, A., Kulisevsky, J., Garcıa-Sánchez, C., Berthier, M., & Estevez-Gonzalez, A. (2003). Effects of repetitive transcranial magnetic stimulation on memory subtypes: a controlled study. Neuropsychologia, 41(14), 1877-1883.
20. Rollnik, J. D., Düsterhöft, A., Däuper, J., Kossev, A., Weissenborn, K., & Dengler, R. (2002). Decrease of middle cerebral artery blood flow velocity after low-frequency repetitive transcranial magnetic stimulation of the dorsolateral prefrontal cortex. Clinical neurophysiology, 113(6), 951-955.
21. Satow, T., Mima, T., Hara, H., Oga, T., Ikeda, A., Hashimoto, N., & Shibasaki, H. (2002). Nausea as a complication of low-frequency repetitive transcranial magnetic stimulation of the posterior fossa. Clinical neurophysiology, 113(9), 1441-1443.
22. Stewart, L., Walsh, V., Frith, U., & Rothwell, J. C. (2001). TMS produces two dissociable types of speech disruption. *Neuroimage*, *13*(3), 472-478.

**dTMS references**

1. de Andrade, D. C., Galhardoni, R., Pinto, L. F., Lancelotti, R., Rosi Jr, J., Marcolin, M. A., & Teixeira, M. J. (2012). Into the island: a new technique of non-invasive cortical stimulation of the insula. *Neurophysiologie Clinique/Clinical Neurophysiology*, *42*(6), 363-368.
2. Fadini, T., Matthäus, L., Rothkegel, H., Sommer, M., Tergau, F., Schweikard, A., ... & Nitsche, M. A. (2009). H-coil: Induced electric field properties and input/output curves on healthy volunteers, comparison with a standard figure-of-eight coil. Clinical Neurophysiology, 120(6), 1174-1182.
3. Gaede, G., Hellweg, R., Zimmermann, H., Brandt, A. U., Dörr, J., Bellmann-Strobl, J., ... & Pfueller, C. F. (2014). Effects of deep repetitive transcranial magnetic stimulation on brain-derived neurotrophic factor serum concentration in healthy volunteers. Neuropsychobiology, 69(2), 112-119.
4. Lenoir, C., Algoet, M., Vanderclausen, C., Peeters, A., Santos, S. F., & Mouraux, A. (2018). Report of one confirmed generalized seizure and one suspected partial seizure induced by deep continuous theta burst stimulation of the right operculo-insular cortex. Brain stimulation, 11(5), 1187.
5. Spagnolo, P. A., Wang, H., Srivanitchapoom, P., Schwandt, M., Heilig, M., & Hallett, M. (2018). Lack of Target Engagement Following Low‐Frequency Deep Transcranial Magnetic Stimulation of the Anterior Insula. Neuromodulation: Technology at the Neural Interface.
6. Zangen, A., Roth, Y., Voller, B., & Hallett, M. (2005). Transcranial magnetic stimulation of deep brain regions: evidence for efficacy of the H-coil. Clinical neurophysiology, 116(4), 775-779.

**tDCS References**

1. Fregni, F., Boggio, P. S., Nitsche, M., Bermpohl, F., Antal, A., Feredoes, E., ... & Pascual-Leone, A. (2005). Anodal transcranial direct current stimulation of prefrontal cortex enhances working memory. Experimental brain research, 166(1), 23-30.
2. Guarienti, F., Caumo, W., Shiozawa, P., Cordeiro, Q., Boggio, P. S., Benseñor, I. M., ... & Brunoni, A. R. (2015). Reducing transcranial direct current stimulation‐induced erythema with skin pretreatment: considerations for sham‐controlled clinical trials. Neuromodulation: Technology at the Neural Interface, 18(4), 261-265.
3. Iyer, M. B., Mattu, U., Grafman, J., Lomarev, M., Sato, S., & Wassermann, E. M. (2005). Safety and cognitive effect of frontal DC brain polarization in healthy individuals. Neurology, 64(5), 872-875.
4. Kessler, S. K., Turkeltaub, P. E., Benson, J. G., & Hamilton, R. H. (2012). Differences in the experience of active and sham transcranial direct current stimulation. Brain stimulation, 5(2), 155-162.
5. Poreisz, C., Boros, K., Antal, A., & Paulus, W. (2007). Safety aspects of transcranial direct current stimulation concerning healthy subjects and patients. Brain research bulletin, 72(4-6), 208-214.
6. Riedel, P., Kabisch, S., Ragert, P., & von Kriegstein, K. (2012). Contact dermatitis after transcranial direct current stimulation. Brain Stimulation: Basic, Translational, and Clinical Research in Neuromodulation, 5(3), 432-434.
7. Tadini, L., El-Nazer, R., Brunoni, A. R., Williams, J., Carvas, M., Boggio, P., ... & Fregni, F. (2011). Cognitive, mood, and electroencephalographic effects of noninvasive cortical stimulation with weak electrical currents. The journal of ECT, 27(2), 134-140.
