# Supplementary material for: A retrospective qualitative report of symptoms and safety from transcranial focused ultrasound for neuromodulation in humans
Source: Sci Rep. 2020 Mar 27;10:5573. doi: 10.1038/s41598-020-62265-8 (PMC7101402; doi:10.1038/s41598-020-62265-8)
Supplement: Supplementary file 2 — Supplementary information2. [file 41598_2020_62265_MOESM2_ESM.pdf]

Subject ID: PI:

Participant Report of Symptoms

Type of Non-Invasive Brain Stimulation: TMS____tFUS_____Other (Describe):______________________

**Visit Date:**		**POST** Time: Investigator:	
**How are you feeling overall right now?**	Participant		
**"Right now, do you feel you have or are……?"**		**Value**<br><br>1 absent<br>2 mild<br>3 moderate<br>4 severe	**Relation**<br><br>1 unrelated<br>2 unlikely<br>3 possible<br>4 probable<br>5 definite
Headache			
Unusual feelings on the skin of your head			
Neck pain			
Tingling			
Itchiness			
Sleepiness			
Difficulty paying attention			
Unusual /feelings, attitude, emotions			
Tooth pain			
Change in hearing			
Nausea/Sick to Stomach			
Unusual twitches or movements in muscles			
Dizziness			
Anxious/Worried/Nervous			
Forgetful			
Difficulty with your balance			
Change in movement in your stronger hand			
Abnormal sleep last night			
Seizure within the last 24 hours			
Other:			
